# Supplementary material for: Lung cancer associated autoantibody responses are detectable years before clinical presentation
Source: PLoS One. 2025 Mar 11;20(3):e0315220. doi: 10.1371/journal.pone.0315220 (PMC11896025; doi:10.1371/journal.pone.0315220)
Supplement: S1 Appendix — (PDF) [file pone.0315220.s001.pdf]

Appendix 1: Longitudinal autoantibody profiles in positive cases

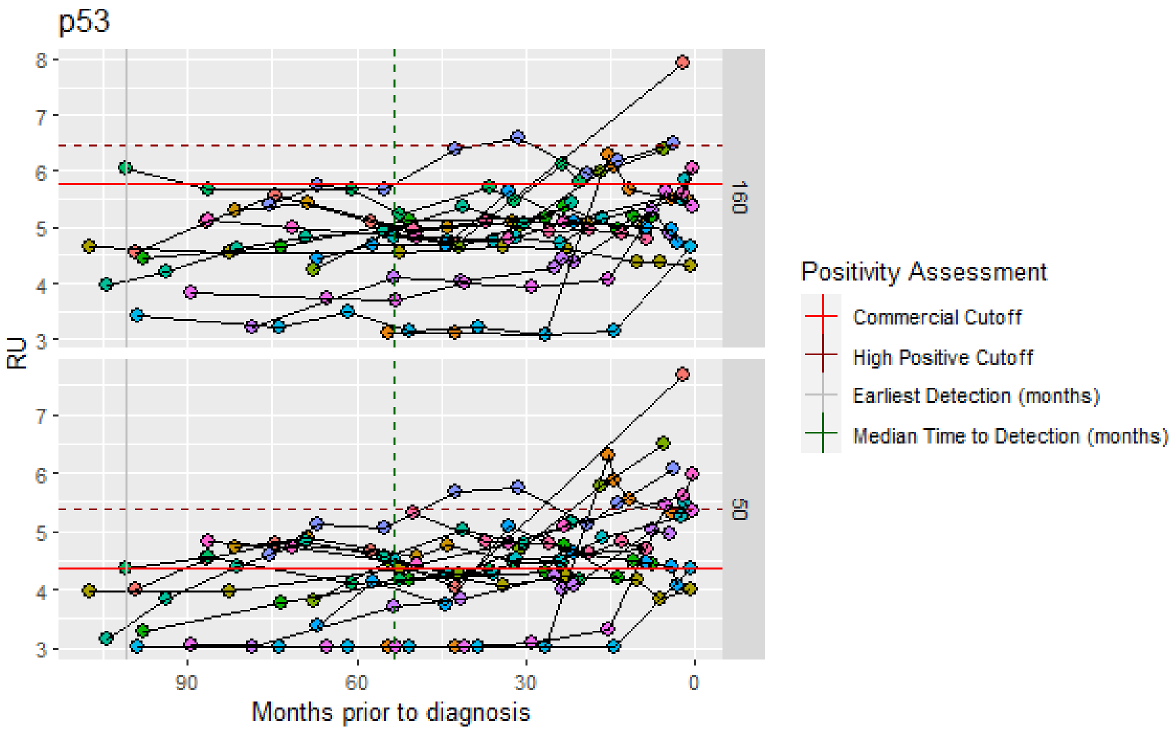

Figure S1-1: Positive longitudinal p53 autoantibody profiles by subject, with earliest positive signal (first patient sample elevated above commercial cutoff), and median time to detection indicated.

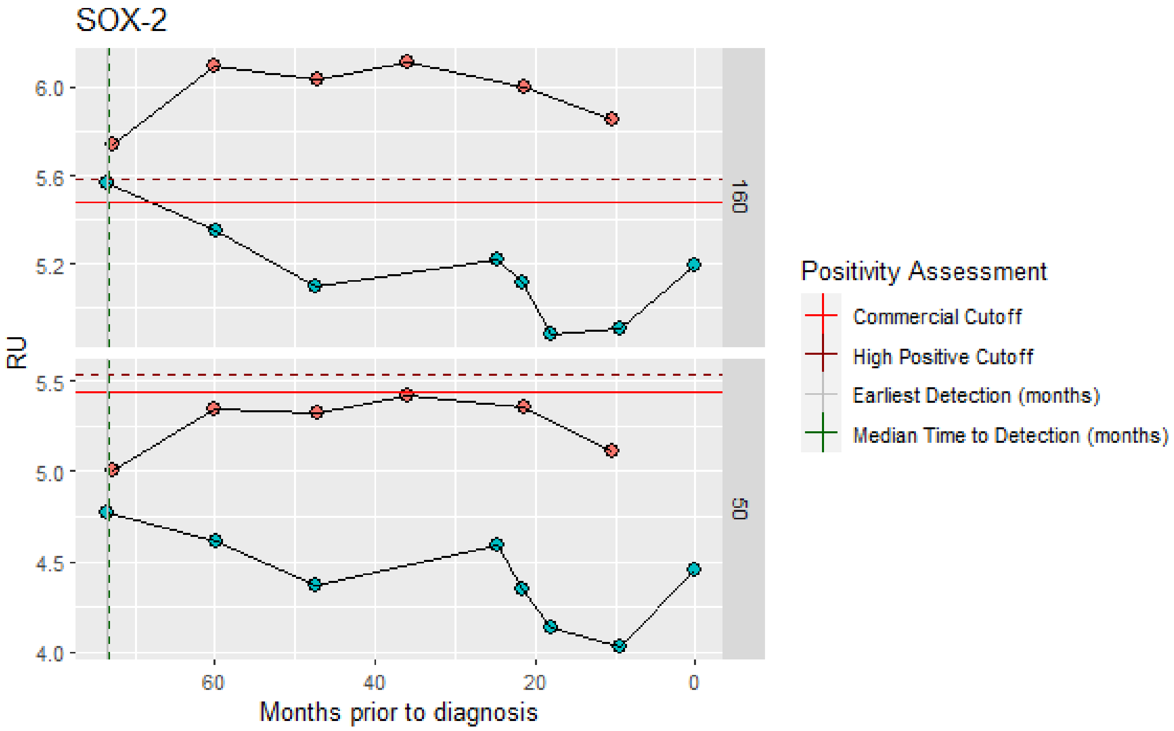

Figure S1-2: Positive longitudinal SOX-2 autoantibody profiles by subject, with earliest positive signal (first patient sample elevated above commercial cutoff), and median time to detection indicated.

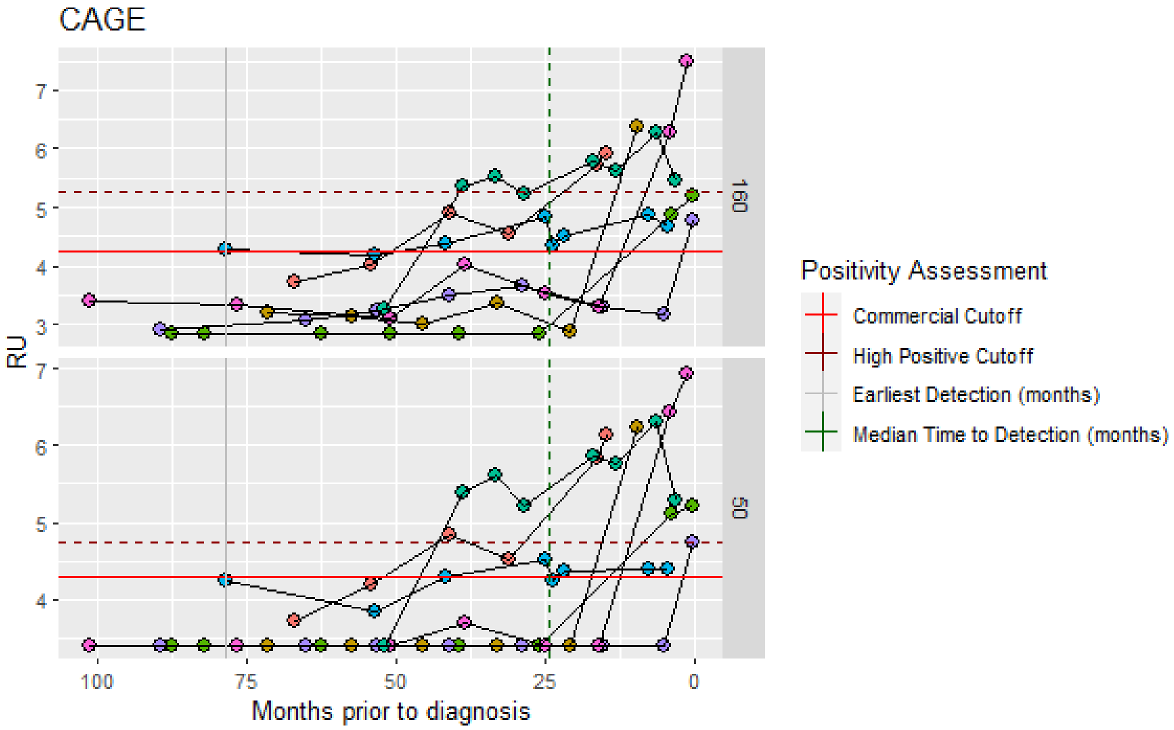

Figure S1-3: Positive longitudinal CAGE autoantibody profiles by subject, with earliest positive signal (first patient sample elevated above commercial cutoff), and median time to detection indicated.

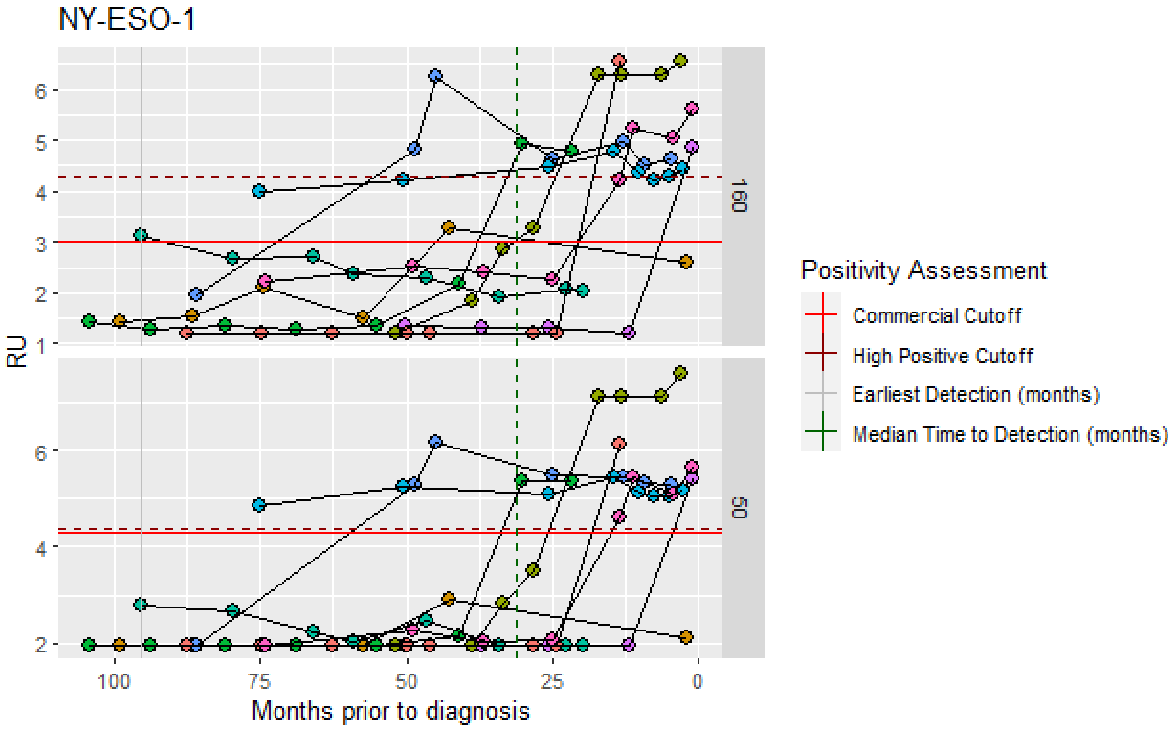

Figure S1-4: Positive longitudinal NY-ESO-1 autoantibody profiles by subject, with earliest positive signal (first patient sample elevated above commercial cutoff), and median time to detection indicated.

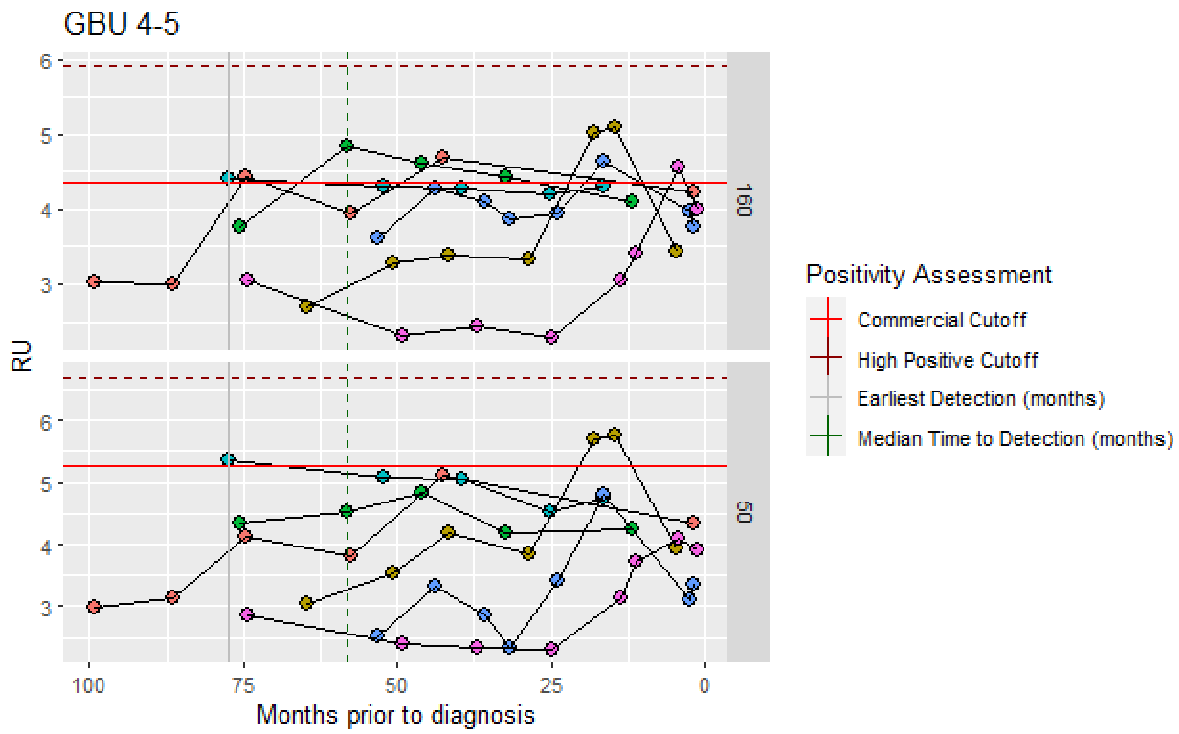

Figure S1-5: Positive longitudinal GBU 4-5 autoantibody profiles by subject, with earliest positive signal (first patient sample elevated above commercial cutoff), and median time to detection indicated.

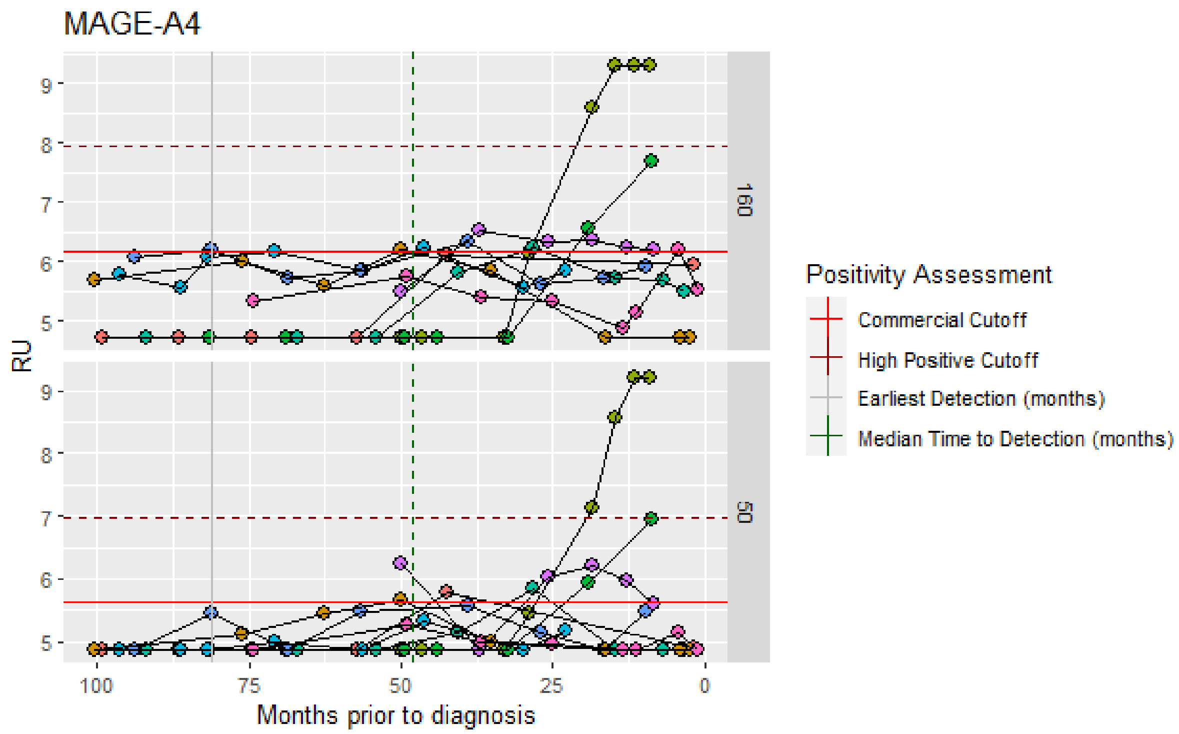

Figure S1-6: Positive longitudinal MAGE-A4 autoantibody profiles by subject, with earliest positive signal (first patient sample elevated above commercial cutoff), and median time to detection indicated.
